# Radiomics Analysis for ^177^Lu-DOTAGA-(l-y)fk(Sub-KuE) Targeted Radioligand Therapy Dosimetry in Metastatic Prostate Cancer—A Model Based on Clinical Example

**DOI:** 10.3390/life11020170

**Published:** 2021-02-22

**Authors:** Eve Kelk, Priit Ruuge, Kristi Rohtla, Anne Poksi, Kalevi Kairemo

**Affiliations:** 1 Center of Nuclear Medicine, East Tallinn Central Hospital, 10138 Tallinn, Estonia; kristi.rohtla@itk.ee (K.R.); anne.poksi@itk.ee (A.P.); 2 Department of Biomedical Engineering, East Tallinn Central Hospital, 10138 Tallinn, Estonia; priit.ruuge@itk.ee; 3 Department of Nuclear Medicine, University of Texas MD Anderson Cancer Center, Houston, TX 77030, USA; kalevi.kairemo@gmail.com

**Keywords:** prostate cancer, PSMA, radionuclide therapy, radiomics, dosimetry, voxel-based dosimetry, ^177^Lu isotope, positron emission tomography, quantitative SPECT

## Abstract

^177^Lu-DOTAGA-(l-y)fk(Sub-KuE) a.k.a. ^177^Lu-PSMA I&T is currently used for radioligand therapy (RLT) of metastatic castration-resistant prostate cancer (mCRPC) in several centers in Europe. Background: Dosimetry is mandatory according to EU guidelines, although routine methods for dosimetry, i.e., absorbed radiation dose calculations for radiopharmaceuticals, are missing. Methods: We created a model of dosimetric analysis utilizing voxel-based dosimetry and intra-lesion radiomics to assess their practicality in routine dosimetry. Results: As an example for the model, our patient with mCRPC had excellent therapy response; quantitatively more than 97% of the metastatic tumor burden in local and distant lymph nodes and skeleton was destroyed by four cycles of RLT. The absorbed radiation doses in metastases decreased towards later cycles of RLT. Besides the change of prostate-specific membrane antigen (PSMA) concentration and absorbed doses in the tumor, further response to RLT could be predicted from biomarker changes, such as LDH and PSA. Conclusions: Individual dosimetry is needed to understand large variations in tumor doses and mixed responses; for that purpose, routine tools should be developed. The Dosimetry Research Tool (DRT) fluently performed automated organ delineation and absorbed radiation dose calculations in normal organs, and the results in our patient were in good concordance with the published studies on ^177^Lu-PSMA dosimetry. At the same time, we experienced considerable challenges in voxel-based dosimetry of tumor lesions. Measurements of ^177^Lu-PSMA activity concentrations instead of absorbed radiation dose calculations could make routine dosimetry more flexible. The first cycle of RLT seems to have quantitatively the biggest impact on the therapy effect. Radiomics analyses could probably aid in the treatment optimization, but it should be tested in large patient populations.

## 1. Introduction

Prostate cancer is the second most common malignancy in men after lung cancer. GLOBOCAN, a project of the International Agency for Research on Cancer, estimated 7.1% of all new cancer cases and 3.8% of cancer-related deaths worldwide to be due to prostate cancer in 2018 [1]. The last decade has brought promising new prostate-specific membrane antigen (PSMA)-related diagnostic and therapeutic tools to the management of prostate cancer [2,3]. PSMA has two enzymatic functions in the body; depending on its site of action, it acts as folate hydrolase I or as glutamate carboxypeptidase II. It is a transmembrane protein, which among other organs is expressed in the membrane of prostate epithelial cells. In prostate cancer, it is 100 to 1000 times upregulated, demonstrating a stronger expression on cancer cells the more aggressive the cancer is [4,5]. That makes PSMA an ideal marker to target prostate cancer cells both for visualization and therapy.

**Figure 1:**
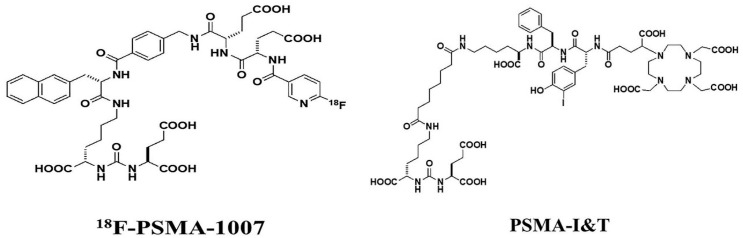
Chemical structure of ^18^F-PSMA-1007 and DOTAGA-(l-y)fk(Sub-KuE), also named PSMA-I&T. The former is a positron-emitting imaging agent and the latter is a therapeutic compound when labeled with beta emitting ^177^Lu. ^177^Lu-atom is coupled with DOTAGA macrocyclic chelate with the PSMA molecule; there is a spacer between the radionuclide part and the binding motif to protect it from radiolysis.

Several large and well-designed studies have been conducted on radioligand therapy (RLT) of metastatic castration resistant prostate cancer (mCRPC) with ^177^Lu-PSMA-617. ^177^Lu-labeled PSMA inhibitor for imaging and therapy (PSMA I&T; Figure 1), a DOTAGA-chelated urea-based PSMA inhibitor developed by Munich Technical University, is less widely studied, yet it has demonstrated equal efficacy with ^177^Lu-PSMA-617 [6]. RLT with ^177^Lu-PSMA ligands has not received standard therapy status so far; it is expected to happen after the prospective multicenter randomized phase 3 study of ^177^Lu-PSMA-617 in mCRPC (VISION) is concluded in 2021 [7].

In East Tallinn Central Hospital (ETCH), RLT has been routinely performed since December 2018. We have witnessed very different patterns of clinical effectiveness of RLT, making us seek for an optimal quantitative tool to assess the distribution of radioactivity which could aid in predicting the outcome of RLT, preferably already after the first cycle of RLT. As part of this process, a radiomics approach was introduced. 

Here, an extensive quantitative analysis of one patient was performed, consisting of four cycles of ^177^Lu-PSMA I&T (Lu-PSMA) using the Dosimetry Research Tool (DRT). We compared the data about different types of lesions—local and distant lymph node metastases and bone metastases—acquired from the voxel-based dosimetry the tool offers, with standardized uptake values (SUV) gained on contemporary quantitative single photon emission computed tomography/computed tomography (SPECT/CT) system. Voxel-based dosimetry is not commonly used in RLT. Our aim was to evaluate if this labor- and time-consuming method would add an extra value in predicting the response to radioligand therapy already after the first cycle of RLT. 

## 2. Results

### 2.1. Clinical Response

75-year-old patient with mCRPC received four cycles of ^177^Lu-DOTAGA-(l-y)fk(Sub-KuE), radioligand therapy with 7–10 weeks intervals, mean administered activity was 7395 MBq (range 6934–7722 MBq).

No immediate side effects occurred during the RLT, also no serious adverse events were detected after any cycle of RLT. Only grade 1 anemia was detected after the 2nd cycle of RLT, that did not become worse after the subsequent therapy cycles. After 2 first cycles of RLT the physical condition of the patient improved from ECOG 1 to 0, he was completely free of complaints.

### 2.2. Biochemical and Molecular Response

Biochemical response was evaluated by comparing the pre- and post-treatment values of PSA. PSA decreased 84.1% in result of the 1st cycle of RLT—from 154.2 µg/L to 24.6 µg/L, corresponding to partial response (PR). At week 6 after the last cycle of RLT PSA has decreased to 1.05 µg/L—99.3% from the initial level. The levels of alkaline phosphatase and lactate dehydrogenase were in normal range before the start of RLT and remained stable throughout the therapy (Figure S1 in Supplementary Materials).

**Figure 2:**
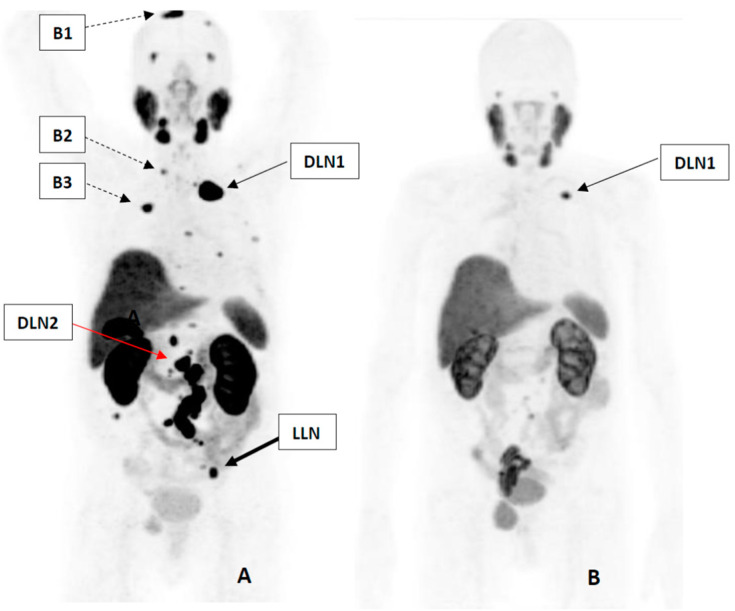
^18^F-PSMA-1007 PET/CT of the patient. (**A**) Baseline scan. Additionally to physiological uptake of the tracer, three types of metastases were detected: a local lymph node metastasis at left in the pelvis (LLN), distant lymph node metastases in left supraclavicular region and around abdominal aorta (DLN1*–*2) and metastases in cortical bone (B1–3). (**B**) Follow-up scan, performed 6 weeks after the 4th cycle of radioligand therapy (RLT). The total tumor burden decreased from 160.3 mL to 4.1 mL (97.4%).

Molecular response was evaluated by comparing the pre- and post-treatment ^18^F-PSMA-1007 positron emission tomography/computed tomography (PET/CT) scans. The highest SUVmax was recorded for both PET studies irrespective of number of lesions (Figure 2). In our case 80.7% drop of SUVmax was achieved, corresponding to PR based both on European organization for research and treatment of cancer (EORTC) criteria and PET response criteria in solid tumors 1.0 (PERCIST 1.0) [8,9]. Although EORTC and PERCIST 1.0 systems are established for evaluating treatment response for ^18^FDG PET/CT, several authors have used both evaluation systems successfully in PSMA PET, showing good concordance with biochemical response exceeding the one of morphological imaging [10,11].

In result of four cycles of RLT total tumor burden, measured with MTV tool of *syngo*. via, decreased 97.4% (from 160.3 mL to 4.1 mL). Furthermore, after completion of our analysis, two additional cycles of RLT were performed. In result of six cycles the total tumor burden decreased to 1.27 mL—99.2% from the initial burden, and PSA decreased to 0.26—99.8% from the pre-treatment level.

### 2.3. Dosimetric Results

**Figure 3:**
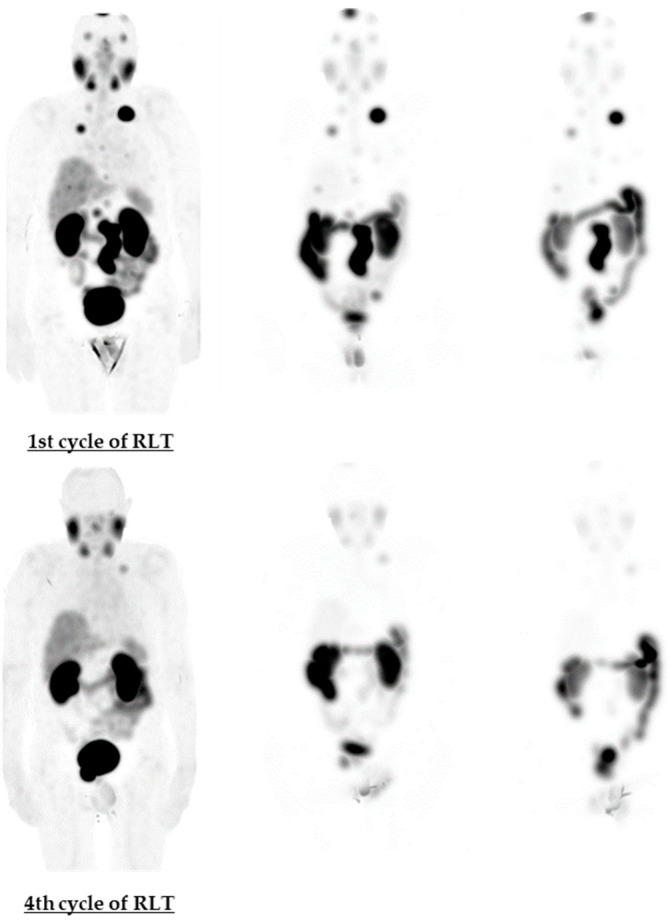
Maximum intensity projection (MIP) images during the 1st and 4th cycle of ^177^Lu-PSMA I&T therapy. From left to right 4, 24 and 48 h images are presented. At the 4th cycle PSMA expression is significantly less expressed in body, both in tumorous tissue, demonstrating its eradication, but also in lacrimal and salivary glands, possibly showing their damage in result of 3 previous cycles of RLT.

Voxel-based and organ-based dosimetry with DRT software [12] were performed from the quantitative SPECT/CT images acquired 4, 24 and 48 h after injection of Lu-PSMA. Illustrative whole-body maximum intensity projection (MIP) images from these time points are shown in Figure 3. The absorbed doses in critical organs (lacrimal and salivary glands, kidneys) and in liver and spleen are presented in Table 1. 

Absorbed-dose map for voxel-based dosimetry was created with matrix size 64 and monoexponential fit between time points. Large organ segmentation was created automatically, but small organs (lacrimal and salivary glands) and metastatic lesions were segmented manually with spherical volume of interest (VOI) large enough to cover visually high dose regions. Since there are uncertainties due to registration errors and partial volume effect, the following equation for compensation of these effects was used:

(1) $$D\left(Gy\right)=\frac{Mean\left(Gy\right)\times Volume\_of\_VOI\left(ml\right)}{Volume\_of\_Organ\left(ml\right)}$$

where D is absorbed dose in organ, Mean is mean dose in spherical VOI, Volume_of_Organ is volume of organ or tumor lesion measured on computed tomography (CT). Since the absorbed doses of the bigger organs (kidneys, spleen, liver) are less influenced by registration errors and partial volume effect, the absorbed dose of those is less underestimated and does not need correction. Voxel size is 1.95 mm × 1.95 mm × 1.95 mm. 

**Figure 4:**
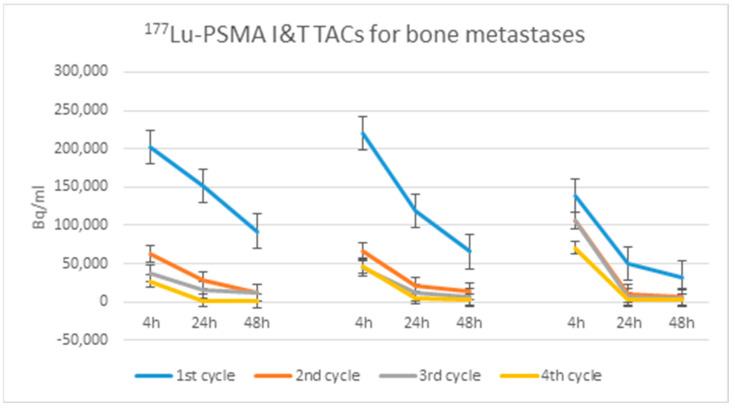
^177^Lu time-activity curves (TAC) in three metastatic sites in the skeleton during four cycles of RLT. The ^177^Lu activity concentration decreases towards later time point and towards later cycles.

Voxel-based absorbed doses were also calculated in three different kinds of metastatic lesions: loco-regional lymph node metastases (below the bifurcation of common iliac arteries), distant lymph node metastases, and bone metastases. The calculated absorbed radiation doses are presented in Table 2, and their volumes and SPECT SUVmax values during the four cycles of RLT in Table 3. The absorbed radiation doses in tumor lesions decrease systematically towards the later RLT cycles (Table 2), and a similar trend is observed in their SUVmax values. Additionally, the volume of all analyzed lesions decreases as a result of RLT (Table 3). Figure 4 demonstrates the dynamic behavior of ^177^Lu activity concentration in three different skeletal metastases of one patient. In later cycles, when the tumor size remains constant, the activity concentration still decreases (Table 3).

## 3. Discussion

Mixed responses in different lesions can be observed during targeted therapies with ^177^Lu-PSMA [13,14,15,16]. Our analysis of this four-cycle RLT series was driven by the interest in single lesion kinetics at voxel level, i.e., intra-lesion radiomics. As the effect on radionuclide therapy depends on the accumulation of the radiopharmaceutical and its residence time in the lesion, voxel-based dosimetric analysis could aid in the better planning of ^177^Lu-PSMA I&T therapies—selection of patients and predicting how much disease is possible to eradicate in a certain case.

We created a model of dosimetric analysis with performing precise 3D measurements on pre- and post-RLT PET/CT scans and on quantitative SPECT/CT scans at three time points of four cycles of RLT. This case is a demonstration of how such analysis should be carried out. The complex data of our example series are presented in Table 2 and Table 3. To draw conclusions on whether such labor- and time-consuming analysis is worth performing for every single RLT patient, this model should be tested on a larger scale.

Marked decrease of tumor doses was observed during the first two cycles of RLT. This is explained by different factors of tumor biology, e.g., death of tumor cells and bigger decrease of viable tumor volume in result of the earlier therapy cycles. Therefore, it has been proposed to administer higher activities during the first or the first two cycles of RLT [17]. Performing accurate dosimetry of the malignant tissue and the critical organs after every cycle of RLT is mandatory to make adjustments to individual therapy plans to safely achieve higher cumulative tumor doses.

It is known that the presence of skeletal metastases is an important prognostic factor in the outcome of ^177^Lu-PSMA RLT [14], whereas the presence of lymph node metastases does not affect the outcome of RLT. In our case, the distant lymph node metastases demonstrated higher absorbed radiation doses compared to metastases in local lymph nodes and the skeleton. On the other hand, there was a large variation in the skeletal metastases, both in absorbed radiation doses (Table 2) and in radiopharmacokinetics (Figure 4). The difference in the mean absorbed dose of the three skeletal lesions was more than 10-fold between the first and the fourth cycle—a reduction from 38.4 Gy to 3.7 Gy was observed. Also, the same lesion could receive more than 10-fold dose in the first cycle as compared to the last or even the third cycle.

It is known from many studies, e.g., from the German multicenter study [15], that typically the highest dose is given by the first cycle. This is also the case here. Already after the first cycle we could, based on our mathematical calculations, predict that this patient would respond well to RLT. The same could also be indirectly predicted from the behavior of biomarkers PSA and LDH. At the same time the extent of this excellent outcome—here more than 97% of the original tumor burden was destroyed by four cycles of RLT, and more than 99% with two additional cycles later—could not be predicted in advance.

Regarding normal organs, our results do not differ significantly from other dosimetry studies with ^177^Lu-PSMA compounds [16,18]. However, direct comparison is not possible, as our model contains 3D, not planar image analysis, and different time points for creating time-activity curves are used. The precision the DRT cannot be properly judged here, because we have only tested it against organ-based dosimetry (medical internal radiation dose, MIRD) in larger organs and SPECT SUVmax in small organs and tumor lesions. The automated organ delineation and absorbed radiation dose calculations were fluently performed by DRT, and the results in our patient were in good concordance with the published studies on ^177^Lu-PSMA dosimetry [16,18,19].

We did not find any correlation between the absorbed dose in a specific lesion and the SUVmax decrease it created on quantitative SPECT/CT (Table 2; Table S2 in Supplementary Materials). This may refer to possible difficulties in individual planning of RLT. 

### The Limitations of the Study

The precision of measuring the absorbed radiation dose in smaller lesions is poor due to the partial volume effect. Phantom studies have shown that SPECT/CT activity quantification uncertainty can be even 18% [20]. Due to spatial registration errors between different time points and lack of an isocontouring option when using manual VOI in DRT, errors for small organs and metastases can be considerably higher. Also, depending on localization of the lesions, “noise” from adjacent organs has strong influence on the measurements—e.g., in our case, the local lymph node in the pelvis, where high uptake of radiopharmaceutical by colon and in the bladder are disturbing the measurements. An additional source of error is measuring the volume of small organs and metastases from CT—especially because the bone metastases are difficult to delineate and measure.

As manually drawn VOIs were used, the time-activity curves (TAC) created by DRT, based on activity concentration (Bq/mL), are not directly comparable with each other, but the slopes of the curves are comparable.

Choosing three time points—4, 24, and 48 h—for measurements is not ideal. Time integrated activity after 48 h cannot be verified in such case. At the same time, looking at the TACs, adding the fourth time point or extending the time between the two last time points does not seem critical for measuring the effective half-life—this is definitely reached within the first 48 h.

The positioning of the patient for all time points is crucial as correcting the differences creates much time-consuming extra work.

In a situation where due to previously mentioned reasons (small, or vice versa, packed in conglomerate metastases; difficult to delineate bone metastases; disturbing activity in adjacent organs) the calculated absorbed doses seem erroneous, biochemical effect (PSA as surrogate marker of response) and information gained on molecular imaging (SPECT SUV change) may be more reliable for making further treatment decisions. Serial quantitative post-therapy SPECT/CT imaging for dosimetry the way we performed it, is too difficult and time-consuming for clinical routine, and too erroneous for previously described reasons. A simplified model, performing only one quantitative SPECT at day 4 (which has been demonstrated to be sufficient to provide a 3D dose map for absorbed doses estimation in radionuclide therapy with ^177^Lu-DOTA peptides) would probably also be a better alternative in RLT [21].

## 4. Materials and Methods

### 4.1. The Patient and ^18^F-PSMA-1007 PET/CT

A 75-year-old patient with mCRPC that progressed under previous standard therapies was treated with 4 cycles of Lu-PSMA. The patient was diagnosed with Gleason 7, G3 prostate adenocarcinoma 9 years earlier, in 2010. He received androgen-deprivation therapy with cyproterone acetate for three years until progression in 2013, after what the surgical castration was performed. Two years later, he needed radiotherapy due to metastatic lymph nodes in his pelvis, and a year later para-aortic lymphadenectomy due to metastatic disease progression. In 2017, bone metastases and lymph node metastases in para-aortic and supraclavicular lymph nodes were revealed and palliative chemotherapy with docetaxel was started. As the disease still continued to progress, a gonadotropin-releasing hormone agonist abiraterone was started. Despite several lines of conventional treatment the patient received, both the level of prostate specific antigen (PSA) and the size of the metastases continued to increase. The PSA doubling time (DT) was two months at the time of evaluation of eligibility for RLT. PSMA expression was demonstrated in loco-regional and distant lymph node metastases and in cortical bone by ^18^F-PSMA-1007 PET/CT (Figure 2). As all other therapeutic options had been used, in concordance with the Helsinki declaration (Art. 37) Lu-PSMA radioligand therapy (RLT) was decided as an additional treatment by the multidisciplinary tumor board; the decision was approved by institutional body. Informed consent was obtained from the patient both for the therapy and for the subsequent analysis of the therapy results.

There were no contraindications for RLT—hematological tests showed that the renal and hepatic function of the patient were within normal range, his physical condition was moderately good, ECOG 1, the only complaint being back pain (5–6 points on visual analogue pain scale). His concomitant conditions—ischemic cardiac disease (suffered myocardial infarction 12 years earlier) and arterial hypertension were well controlled with appropriate medication.

PET/CT with ^18^F-PSMA-1007 (MAP Medical Technologies OY, Tikkakoski, Finland) was performed 3 weeks before the first cycle of RLT and 6 weeks after the fourth cycle of RLT. Both scans were acquired with GE Medical Systems Discovery STE PET/CT. Imaging started 120 min after intravenous administration of ^18^F-PSMA-1007, activity 210–240 MBq. PET image from the skull through mid-thigh in 3D flow motion was acquired together with low-dose CT-scans from the same region. Maximum standardized uptake values (SUVmax) were obtained by drawing circular regions of interest, which were automatically adapted (40% isocontour) to a 3D volume of interest (VOI) using commercial software *syngo*.via by Siemens Medical Solutions USA, Inc., Malvern, PN, USA. Total tumor burden was calculated from both scans, using Metabolic Tumor Volume (MTV) tool, SUV threshold 3.5.

### 4.2. Manufacturing, Administration and Side Effects of Lu-PSMA

^177^Lu-DOTAGA-(l-y)fk(Sub-KuE) a.k.a. ^177^Lu-PSMA-I&T is a radiopharmaceutical for compassionate use in European Union. The product was provided to ETCH by MAP Medical Technologies Oy, Tikkakoski, Finland under a special license issued by the Estonian State Agency of Medicine (number of primary permission T47264/92783). Quality control including radiochemical identity, radiochemical purity, pH, ascorbic acid concentration, ^177^Lu-PSMA I&T concentration and specific activity, endotoxin content and sterility performed by MAP Medical Technologies Oy, Tikkakoski, Finland.

Cooling of salivary glands and intravenous infusion of physiological saline solution was started 30 min before injection of Lu-PSMA, and continued for 4 h post injection of the radioligand. Lu-PSMA was administered intravenously during 10 min by syringe infusion pump.

The patient received 4 cycles of RLT with 7–10 week intervals, mean administered activity 7395 MBq (range 6934–7722 MBq).

No immediate side effects occurred during the RLT; also, no serious adverse events were detected after any cycle of RLT. Only grade 1 anemia was detected after the 2nd cycle of RLT, but it did not become worse after the subsequent therapy cycles. After the first 2 cycles of RLT, the physical condition of the patient improved to ECOG 0 and he was free of complaints.

### 4.3. Equipment and Study Protocols

Lu-PSMA images were acquired with SPECT/CT hybrid system Symbia Intevo Bold™ (Siemens Healthcare GmbH, Erlangen, Germany). Whole body scintigraphy was performed 0.5 h after injection and quantitative SPECT/CT (medium energy low penetration collimators, 60 views, time per view 15 s, continuous mode) from top of the head to mid-thigh was acquired 4, 24, and 48 h after injection. Quantitative SPECT reconstruction using xSPECT Quant™ (Broad Quantification) software with standardized CT attenuation, scatter and resolution recovery corrections was performed. Quantitative data about absolute tracer concentration in the body expressed in standardized uptake values (SUV), activity concentration (kBq/mL) or fraction of uptake (%) were acquired. The Dosimetry Research Tool software together with technical support was provided by Siemens Medical Solutions, USA, Inc., Hoffman Estates, IL, USA.

### 4.4. Image Review

Both the PET/CT and the SPECT/CT studies were reviewed by two independent readers. In addition to physiological uptake in lacrimal and salivary glands, intestines, kidneys, liver, and spleen, three different kinds of metastatic lesions were revealed in the body: loco-regional lymph node metastases (below the bifurcation of common iliac arteries), distant lymph node metastases, and bone metastases. The maximum standardized uptake values (SUVmax) for six target lesions (Figure 2) were measured on all acquired PET/CT and SPECT/CT scans.

## 5. Conclusions

Individual dosimetry is needed to understand large variations in tumor doses and mixed responses, and for that purpose, routine tools should be developed. DRT fluently performed automated organ delineation and absorbed radiation dose calculations in normal organs, and the results in our patient were in good concordance with the published studies on ^177^Lu-PSMA dosimetry. At the same time, we experienced considerable challenges in voxel-based dosimetry of tumor lesions. Measurements of ^177^Lu-PSMA activity concentrations instead of absorbed radiation dose calculations could make routine dosimetry more flexible. The first cycle of RLT seems to have quantitatively the biggest impact on the therapy effect. Radiomics analyses could probably aid in the treatment optimization, but it should be tested in large patient populations first.

## Figures and Tables

**Table 1:** Absorbed Dose—Voxel-based and MIRD—in critical and normal organs through 4 cycles of RLT. For small organs (salivary and lacrimal glands, presented as an average of a pair) voxel-based absorbed doses compensated for partial volume effect were calculated. For the rest of organs voxel-based mean absorbed doses and organ-based doses (MIRD) were calculated.

No of RLT Cycle	Method	Right Kidney	Left Kidney	Liver	Spleen	Parotid Glands	Submandibular Glands	Lacrimal Glands
		Gy/GBq						
1st	Voxel	0.31	0.30	0.03	0.01	0.11	0.24	0.80
	MIRD	0.36	0.35	0.03	0.03			
2nd	Voxel	0.39	0.35	0.03	0.03	0.12	0.20	0.51
	MIRD	0.42	0.40	0.04	0.04			
3rd	Voxel	0.37	0.34	0.03	0.03	0.11	0.04	0.37
	MIRD	0.40	0.39	0.04	0.03			
4th	Voxel	0.43	0.36	0.04	0.05	0.12	0.21	0.30
	MIRD	0.47	0.42	0.05	0.05			

**Table 2:** Voxel-based absorbed doses (Gy/GBq) in different types of metastases during 4 cycles of RLT.

Cycle of RLT	LLN	DLN1	DLN2	B1	B2	B3
	Gy/GBq					
1st	6.89	25.57	6.8	5.55	6.25	3.75
2nd	6.03	9.43	3.04	0.64	1.85	1.96
3rd	2.84	8.3	2.59	0.48	0.97	1.86
4th	2.72	7.24	2.91	N/A	N/A	N/A

LLN—local lymph node metastasis below bifurcation of the left common iliac artery, pre-therapy volume 0.67 mL, totally disappeared after 2 cycles of RLT, marked as not applicable (N/A) in the table thereafter; DLN1—distant lymph node metastasis in the left supraclavicular region, decreased from 3.9 mL to 0.5 mL after 4 cycles of RLT; DLN2—distant lymph node metastasis in para-aortal region at the level of left renal artery; B1—metastasis in the right parietal bone; B2—metastasis in the 1st rib at right; B3—metastasis in the 6th rib at right.

**Table 3:** Single photon emission computed tomography (SPECT) SUVmax values in metastases and their volumes during 4 cycles of RLT. Abbreviations are defined in Table 2.

Cycle of RLT		LLN	DLN1	DLN2	B1	B2	B3
1st; Volume (mL)		0.67	3.9	0.6	1.5	0.4	0.3
SUVmax at	4 h	2.69	33.56	24.73	9.5	5.72	2.9
	24 h	2.64	14.36	11.2	4.21	2.33	0.96
	48 h	2.06	9.8	8.9	2.61	1.56	0.66
2nd; Volume (mL)		0.37	1.22	0.4	1.5	0.4	0.3
SUVmax at	4 h	1.14	5.73	3.56	1.83	1.23	1.32
	24 h	0.73	2.28	1.56	0.92	0.42	0.27
	48 h	0.68	1.43	1.28	0.64	0.29	0.15
3rd; Volume (mL)		0.1	0.82	0.26	1.5	0.4	0.3
SUVmax at	4 h	N/A	3.12	N/A	1.02	N/A	N/A
	24 h	N/A	1.19	0.94	0.52	N/A	N/A
	48 h	N/A	0.76	N/A	0.35	N/A	N/A
4th; Volume (mL)		0.1	0.5	0.13	1.5	0.4	0.3
SUVmax at	4 h	N/A	1.96	N/A	0.66	N/A	N/A
	24 h	N/A	0.79	0.5	0.32	N/A	N/A
	48 h	N/A	0.52	N/A	0.22	N/A	N/A

## Supplementary Materials

The following are available online at https://www.mdpi.com/2075-1729/11/2/170/s1, Figure S1: Dynamics of biochemical markers—PSA, ALP and LDH—during the RLT, Table S1: Size of normal and Critical Organs, Table S2: SPECT SUVmax and its decrease (%) in different types of metastases during RLT.

## Abbreviations

ALP	alkaline phosphatase
CT	computed tomography
DRT	Dosimetry Research Tool
ECOG	*Eastern Cooperative Oncology Group* Performance Status
ETCH	East Tallinn Central Hospital
LDH	lactate dehydrogenase
mCRPC	metastatic castration resistant prostate cancer
MIP	maximum intensity projection
MTV	metabolic tumor volume
N/A	not applicable
PET/CT	positron emission tomography/computed tomography
PR	partial response
PSA	prostate specific antigen
PSMA	prostate specific membrane antigen
PSMA I&T	DOTAGA-(l-y)fk(Sub-KuE)
RLT	radioligand therapy
SPECT	single photon emission computed tomography
SPECT/CT	single photon emission computed tomography/computed tomography
SUV	standardized uptake value
SUVmax	maximum standardized uptake value
TAC	time-activity curve
VOI	volume of interest
