# Supplementary material for: Radiomics Analysis for 177Lu-DOTAGA-(l-y)fk(Sub-KuE) Targeted Radioligand Therapy Dosimetry in Metastatic Prostate Cancer—A Model Based on Clinical Example
Source: Life (Basel). 2021 Feb 22;11(2):170. doi: 10.3390/life11020170 (PMC7926837; doi:10.3390/life11020170)
Supplement: Supplementary file 1 [file life-11-00170-s001.pdf]

life

MDPI

Case Report

Radiomics Analysis for ^{177}Lu-DOTAGA-(l-y)fk(Sub-KuE) Targeted Radioligand Therapy Dosimetry in Metastatic Prostate Cancer—A Model Based on Clinical Example

Eve Kelk [1,*], Priit Ruuge [2], Kristi Rohtla [1], Anne Poksi [1] and Kalevi Kairemo [3]

1 Center of Nuclear Medicine, East Tallinn Central Hospital, Tallinn 10138, Estonia; kristi.rohtla@itk.ee (K.R.); anne.poksi@itk.ee (A.P.)
2 Department of Biomedical Engineering, East Tallinn Central Hospital, Tallinn 10138, Estonia; priit.ruuge@itk.ee
3 Department of Nuclear Medicine, University of Texas MD Anderson Cancer Center, Houston 77030, TX, USA; kalevi.kairemo@gmail.com
* Correspondence: eve.kelk@itk.ee

Supplementary Materials

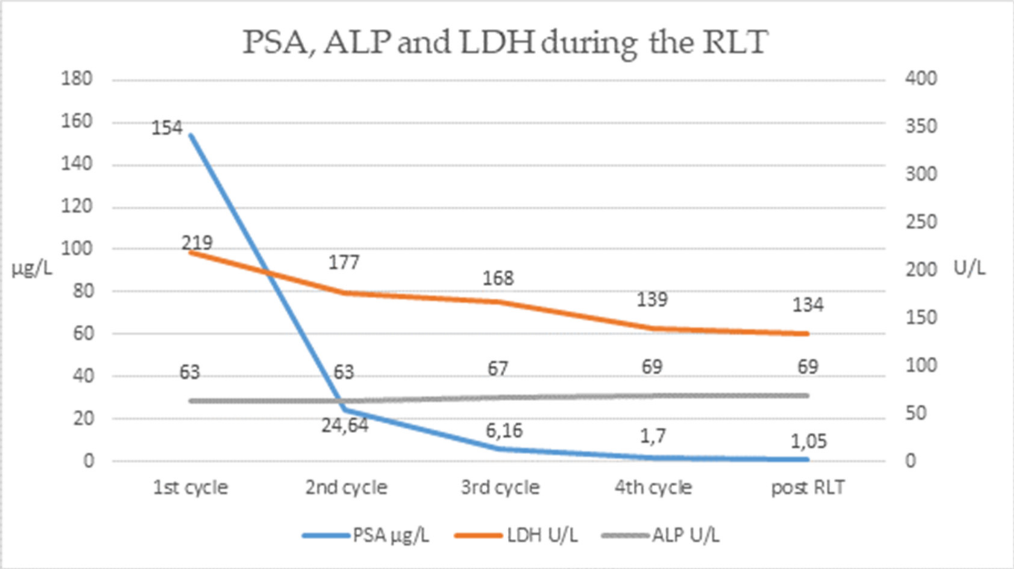

Figure S1. Dynamics of biochemical markers – PSA, ALP and LDH – during the RLT.

Table S1. Size of normal and Critical Organs.

Size of Normal Organs		
	**Voxel-based analysis with DRT** Mean±SD (ml)	**MIRD** Mean±SD (g)
Left Kidney	134.9 ± 3.2	140.4 ± 3.3
Right Kidney	130.6 ± 4.2	136.1 ± 4.4
Liver	1478.9 ± 95.3	1585.9 ± 103.3
Spleen	80.6 ± 7.3	85.1 ± 7.6

Volume of parotid and submandibular glands was measured from CT, it was 23 ml and 5.7 ml respectively. The volume of normal lacrimal glands in Caucasian men – 0.68 ml – found from the literature [1].

Table S2. SPECT SUVmax and its decrease (%) in different types of metastases during RLT.

Lesion	Cycle of RLT	SUVmax at 4h	SUVmax at 24h	SUVmax at 48h
**LLN**	1st	2.69	2.64	2.06
	2nd	1.14	0.73	0.68
**Decrease from 1st to 2nd cycle (%)**		57.6	72.4	67.0
	3rd	disappeared	disappeared	disappeared
**Decrease from 1st to 3rd cycle (%)**		100	100	100
**DLN1**	**1st**	33.56	14.36	9.8
	2nd	5.73	2.28	1.43
**Decrease from 1st to 2nd cycle (%)**		82.9	84.1	85.4
	3rd	3.12	1.19	0.76
**Decrease from 1st to 3rd cycle (%)**		90.7	91.7	92.2
	4th	1.96	0.79	0.52
**Decrease from 1st to 4th cycle (%)**		94.2	94.5	94.7
**DLN2**	1st	24.73	11.2	8.9
	2nd	3.56	1.56	1.28
**Decrease from 1st to 2nd cycle (%)**		85.6	86.1	85.6
	3rd	disappeared	0.94	disappeared
**Decrease from 1st to 3rd cycle (%)**		100	91.6	100
	4th	disappeared	0.5	disappeared
**Decrease from 1st to 4th cycle (%)**		100	100	95.5
**B1**	**1st**	9.5	4.21	2.61
	2nd	1.83	0.92	0.64
**Decrease from 1st to 2nd cycle (%)**		80.74	78.15	75.48
	3rd	1.02	0.52	0.35
**Decrease from 1st to 3rd cycle (%)**		89.26	87.65	86.6
	4th	0.66	0.32	0.22
**Decrease from 1st to 4th cycle (%)**		93.05	92.4	91.57
**B2**	1st	5.72	2.33	1.56
	2nd	1.23	0.42	0.29
**Decrease from 1st to 2nd cycle (%)**		78.5	81.9	81.4
	3rd	disappeared	disappeared	disappeared
**Decrease from 1st to 3rd cycle (%)**		100	100	100
**B3**	1st	2.9	0.96	0.66
	2nd	1.32	0.27	0.15
**Decrease from 1st to 2nd cycle (%)**		54.5	71.9	77.3
	3rd	disappeared	disappeared	disappeared
**Decrease from 1st to 3rd cycle (%)**		100	100	100

References

1. Bingham, C.M.; Castro, A.; Realini, T.; Nguyen, J.; Hogg, J.P.; Sivak-Callcott, J.A. Calculated CT volumes of lacrimal glands in normal Caucasian orbits. *Ophthalmic Plast. Reconstr. Surg.* **2013**, *29*, 157–159, doi:10.1097/IOP.0b013e3182859751.
